# PCP4/PEP19 upregulates aromatase gene expression via CYP19A1 promoter I.1 in human breast cancer SK-BR-3 cells

**DOI:** 10.18632/oncotarget.25651

**Published:** 2018-07-03

**Authors:** Kie Honjo, Taiji Hamada, Takuya Yoshimura, Seiya Yokoyama, Sohsuke Yamada, Yan-Qin Tan, Lai K. Leung, Norifumi Nakamura, Yasuyo Ohi, Michiyo Higashi, Akihide Tanimoto

**Affiliations:** ^1^ Department of Oral Surgery, Kagoshima University Graduate School of Medical and Dental Sciences, Kagoshima, Japan; ^2^ Department of Pathology, Kagoshima University Graduate School of Medical and Dental Sciences, Kagoshima, Japan; ^3^ Department of Pathology and Laboratory Medicine, Kanazawa Medical University, Ishikawa, Japan; ^4^ Faculty of Science, School of Life Sciences, Food and Nutritional Science Programme, The Chinese University of Hong Kong, Shatin, Hong Kong; ^5^ Department of Pathology, Sagara Hospital, Social Medical Corporation Hakuaikai, Kagoshima, Japan

**Keywords:** PCP4/PEP19, breast cancer, aromatase, intratumoral heterogeneity

## Abstract

The Purkinje cell protein 4/peptide 19 (PCP4/PEP19) is a novel breast cancer cell expressing peptide, originally found in the neural cells as an anti-apoptotic factor, could inhibit cell apoptosis and enhance cell migration and invasion in human breast cancer cell lines. The expression of PCP4/PEP19 is induced by estrogens in estrogen receptor-positive (ER^+^) MCF-7 cells but also highly expressed in ER^-^ SK-BR-3 cells. In this study, we investigated the effects of PCP4/PEP19 on aromatase gene expression in MCF-7 and SK-BR-3 human breast cancer cells. In SK-BR-3 cells but not in MCF-7 cells, PCP4/PEP19 knockdown by siRNA silencing decreased the aromatase expression in gene transcriptional level. When PCP4/PEP19 was overexpressed by CMV promoter-driven PCP4/PEP19 expressing plasmid transfection, aromatase gene transcription increased in SK-BR-3 cells. This aromatase gene transcription is mainly mediated through promoter region PI.1, which is usually active in the placental tissue but not in the breast cancer tissue. These results indicate a new function of PCP4/PEP19 that would enhance aromatase gene upregulation to supply estrogens in heterogeneous cancer microenvironment.

## INTRODUCTION

Aromatase is an enzyme, catalyzing the last and rate-limiting step of biosynthesis for estrogens, and expressed in a wide variety of tissues including placenta, ovary, adipose and breast tissues [1]. In the breast cancer tissues, aromatase is expressed in both cancer cells and stromal cells, supplying estrogens locally for breast cancer cells to proliferate in autocrine and paracrine manners, and play a critical role in cancer progression and recurrence [2, 3]. Thus, an aromatase inhibitor is one of therapeutic choices for chemotherapy, especially in estrogen receptor positive (ER^+^) breast cancers of post-menopausal patients [4–6]. Therefore, the understanding of regulation of aromatase gene expression in breast cancer tissues is important to develop a new therapeutic strategy. The aromatase is encoded by cytochrome P450, family 19, subfamily A, polypeptide 1 (*CYP19A1*) gene, and the expression is regulated by several promoter regions classified promoters I and II (PI and PII), which are active in tissue-specific manner [7, 8]. For example, the PI.1, PI.3, PI.4 and PII promoter regions are specifically active in the breast cancer and the region PI.1 and PI.2 promoter works in the placenta [9], while the promoter regions PI.3, PI.4 and PII are active in the adipose tissues [10].

On the other hand, some studies showed that the Purkinje cell protein 4/peptide 19 (PCP4/PEP19) is expressed in the human breast cancer tissues as an estrogen target gene [11–13]. Initially, PCP4/PEP19 has been found to be expressed in the Purkinje cells and inhibits neuronal apoptosis [14]. Previous our studies demonstrated that PCP4/PEP19 is expressed during chemically-induced and estrogen-dependent carcinogenesis in rat mammary gland [15]. The PCP4/PEP19 is an estrogen-inducible peptide and expressed in the human breast cancer tissues and breast cancer cell lines. In ER^+^ MCF-7 cells, the PCP4/PEP19 expression is induced by 17β-estradiol (E2). In contrast, the PCP4/PEP19 is constitutively expressed in a higher level in ER-negative (ER^-^) SK-BR-3 cells [13]. The PCP4/PEP19 expression enhances tumor survival by its anti-apoptotic effect and increased activities of migration and invasion in human breast cancer cell lines [13, 16]. Recently, a new function of the PCP4/PEP19 has been reported that PCP4/PEP19 is expressed in the adrenal cortical tissue and upregulates the expression of aldosterone synthase, encoded by *CYP11B2* gene [17]. Since both aromatase and aldosterone synthase are one of CYP family of steroid hormone biosynthesis, we have an idea that the PCP4/PEP19 may function in the gene regulation of aromatase expression in the human breast cancer cells.

In this study, the regulation of aromatase expression and transcription by PCP4/PEP19 was investigated in MCF-7 and SK-BR-3 human breast cancer cells using promoter specific quantitative RT-PCR and luciferase reporter assay. In SK-BR-3 cells, but not in MCF-7 cells, knockdown of the PCP4/PEP19 decreased aromatase mRNA expression and enzyme activity. Luciferase promoter assay using PI.1, PI.3/II and PI.4 region demonstrated that the PCP4/PEP19 utilizes PI.1 promoter to enhance the aromatase gene transcription in SK-BR-3 cells. In addition to anti-apoptotic, migratory and invasive activities of the PCP4/PEP19 [13, 16], our results indicated the PCP4/PEP19 enhances aromatase expression in ER^-^ SK-BR-3 human breast cancer cells.

## RESULTS

### ER and PCP/PEP19 expression

**Figure 1:**
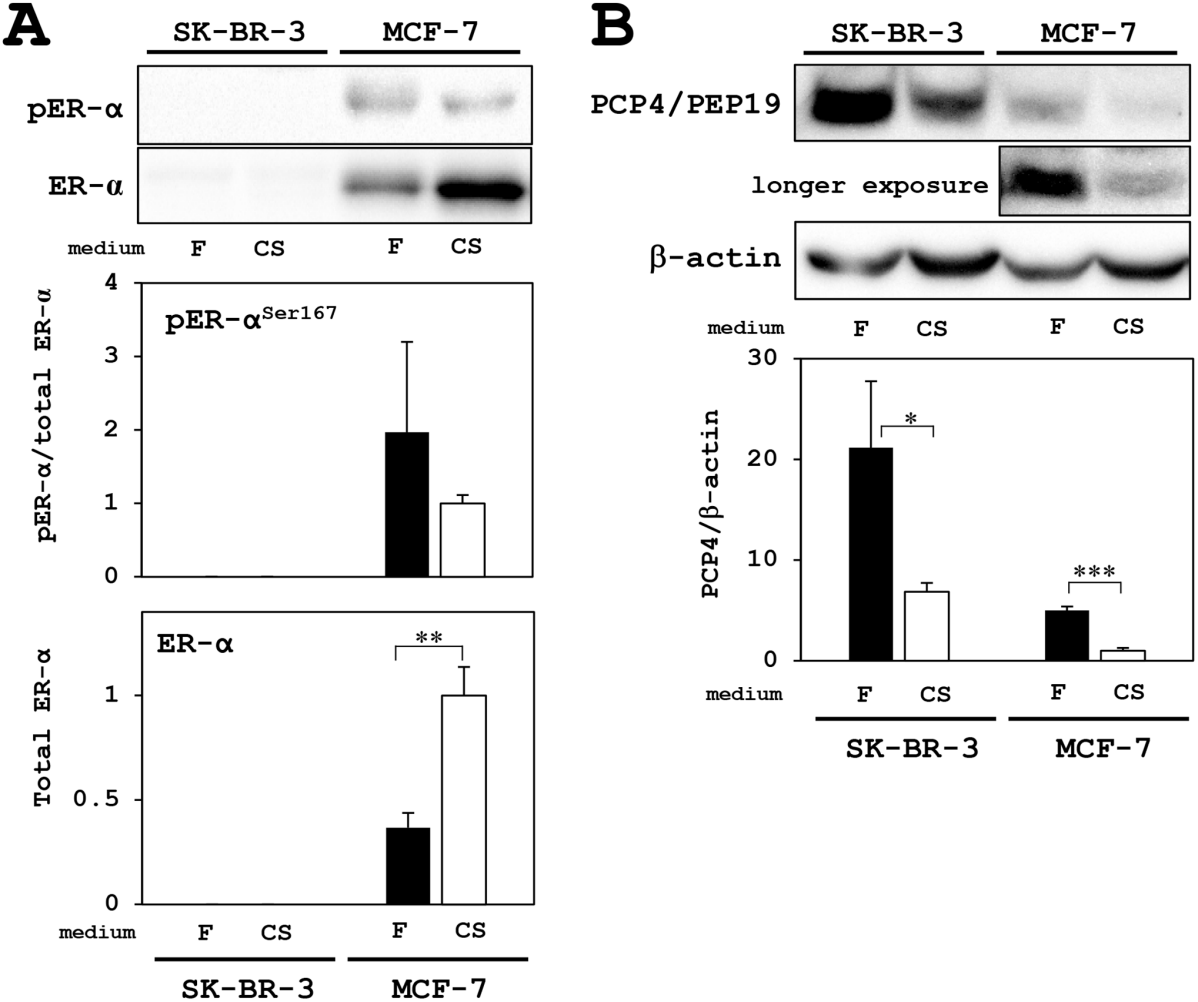
Western blotting analysis for PCP4/PEP19 and ER expression in MCF-7 and SK-BR-3 cells **(A)** ER and active phosphorylated-ER were only detected in MCF-7 cells but not in SK-BR-3 cells. Although total ER-α expression was increased in the culture with charcoal-stripped FBS supplemented medium (CS: open column), the expression levels phosphorylated-ER-α (pER-α) relative to total ER-α were not different in both culture conditions; FBS (F: closed column) and charcoal-stripped FBS (CS: open column) supplementation in the medium. **(B)** PCP4/PEP19 was highly expressed in SK-BR-3 cells, but not in MCF-7 cells, and the expression was decreased in the culture with CS medium (upper blot). In MCF-7 cells, longer exposure revealed PCP4/PEP19 bands, which were decreased in the culture with CS medium (middle blot). Expression levels of PCP4/PEP19 were normalized by those of β-actin and compared between culture conditions (F: FBS as closed column, CS: charcoal-stripped FBS as open column). ^*^ p<0.05, ^**^ p<0.01 and ^***^ p<0.001.

SK-BR-3 human breast cancer cells, in our experimental conditions, expressed no ER-α and phosphorylated ER-α^S167^ even in FBS supplemented medium. In contrast, MCF-7 cells expressed higher levels of both ER-α and phosphorylated ER-α Total ER-α expression was increased after culture with charcoal-stripped FBS (CSFBS) supplemented medium, but phosphorylated fraction of ER-α relative to total ER-α expression levels were not different in both culture conditions (Figure 1A).

In SK-BR-3 cells, PCP4/PEP19 is highly expressed in FBS supplemented culture medium, and the expression was decreased in culture with CSFBS supplemented medium, which showed still higher expression level than in MCF-7 cells. Since PCP4/PEP19 expression was E2 inducible in ER^+^ MCF-7 cells, the basal expression of PCP4/PEP19, in the culture medium with FBS without E2, was very low in comparison with those in SK-BR-3 cells (upper blot in Figure 1B). When the blotting membrane was exposed longer, it revealed PCP4/PEP19 expression, which was decreased in the culture medium with CSFBS (middle blot in Figure 1B). The decreased expression of PCP4/PEP19 may be due to depletion of non-estrogenic steroids as well as due to that of estrogenic steroids or unknown factor(s) which would be stripped by charcoal. We should say that SK-BR-3 cells no longer constitutively express PCP4/PEP19 [13], even the expression in SK-BR-3 cells is still very much higher than in MCF-7 cells.

### Evaluation of siRNA knockdown and plasmid-oriented overexpression of PCP4/PEP19 in SK-BR-3 and MCF-7 cells

**Figure 2:**
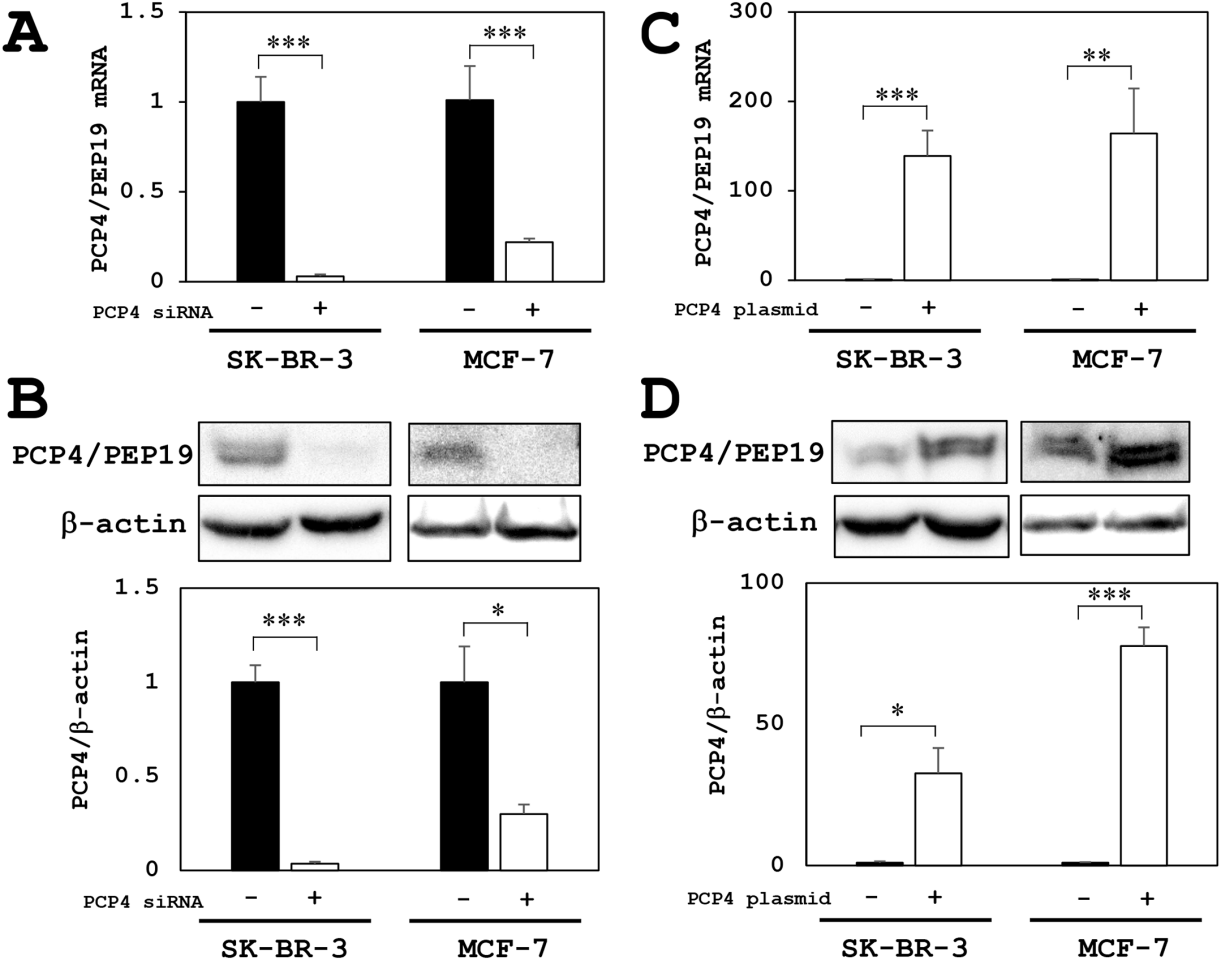
PCP4/PEP19 knockdown and overexpression in MCF-7 and SK-BR-3 cells **(A)** PCP4/PEP19 knockdown by siRNA transfection (PCP4 siRNA + as open column) resulted in markedly decreased PCP4/PEP19 mRNA expression in MCF-7 and SK-BR-3 cells. **(B)** In the same knockdown condition, protein expression of PCP4/PEP19 was significantly decreased in both cell lines. **(C** and **D)** PCP4/PEP19 expressing plasmid transfection (PCP4 plasmid + as open column) showed a significantly increased expression levels of PCP4/PEP19 mRNA (C) and protein (D) in MCF-7 and SK-BR-3 cells. Protein expression levels of PCP4/PEP19 were normalized by those of β-actin. ^*^p<0.05, ^**^ p<0.01 and ^***^ p<0.001.

For experiments, SK-BR-3 cells were cultured in 10% FBS supplemented medium and MCF-7 cells were cultured in 5% charcoal-stripped FBS. By PCP4/PEP19 mRNA silencing by siRNA, the mRNA and protein expression was markedly decreased in SK-BR-3 and MCF-7 cells (Figure 2A and 2B). When CMV promoter-driven PCP4/PEP19 expression plasmid vector was transfected, the mRNA and protein expression was markedly increased in SK-BR-3 and MCF-7 cells (Figure 2C and 2D). Since the siRNA silencing and plasmid-oriented overexpression of PCP4/PEP19 worked very well in both MCF-7 and SK-BR-3 cells, all the experiments afterward studying the PCP4/PEP19 effects on aromatase expression were performed in these experimental conditions.

### Effects of PCP4/PEP19 knockdown and overexpression on aromatase mRNA expression in SK-BR-3 and MCF-7 cells

**Figure 3:**
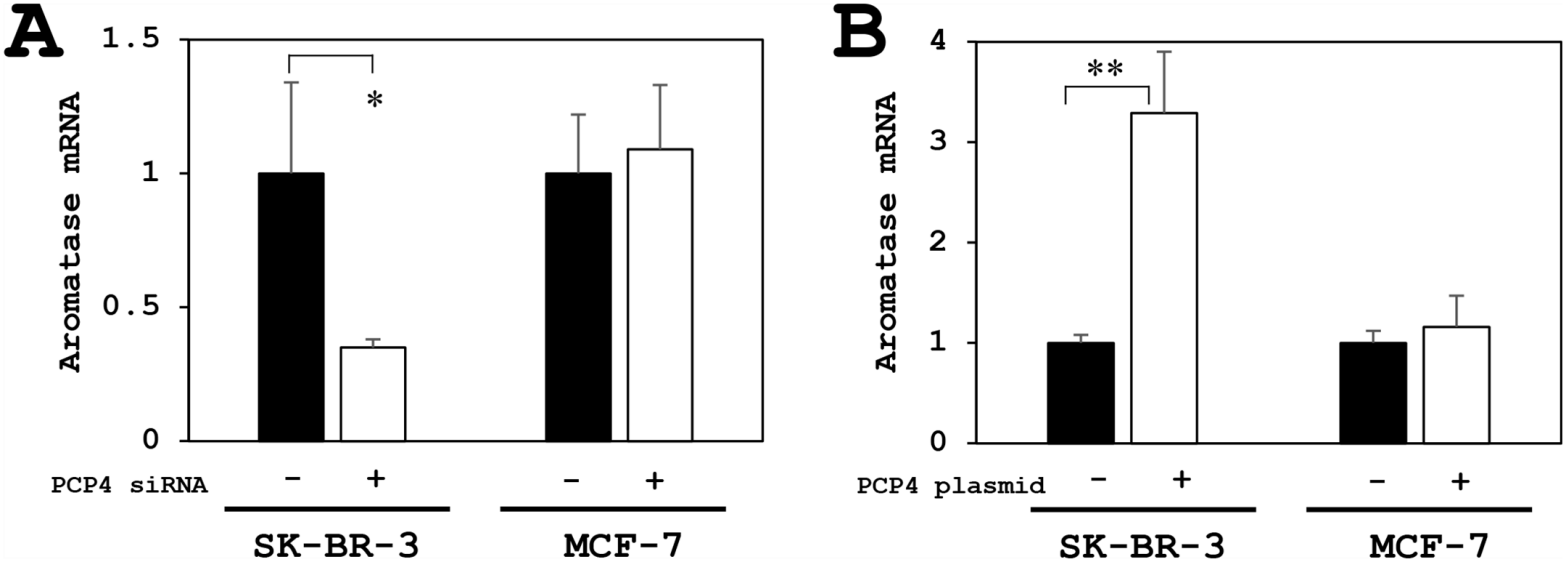
PCP4/PEP19 knockdown and overexpression effects on aromatase mRNA expression in MCF-7 and SK-BR-3 cells **(A)** The aromatase mRNA expression was reduced only in SK-BR-3 cells but not in MCF-7 cells after PCP4/PEP19 knockdown (PCP4 siRNA + as open column). **(B)** Conversely, PCP4/PEP19 overexpression (PCP4 plasmid + as open column) enhanced the aromatase mRNA expression in SK-BR-3 but not in MCF-7 cells. ^*^p<0.05 and ^**^ p<0.01 vs without knockdown or overexpression

**Figure 4:**
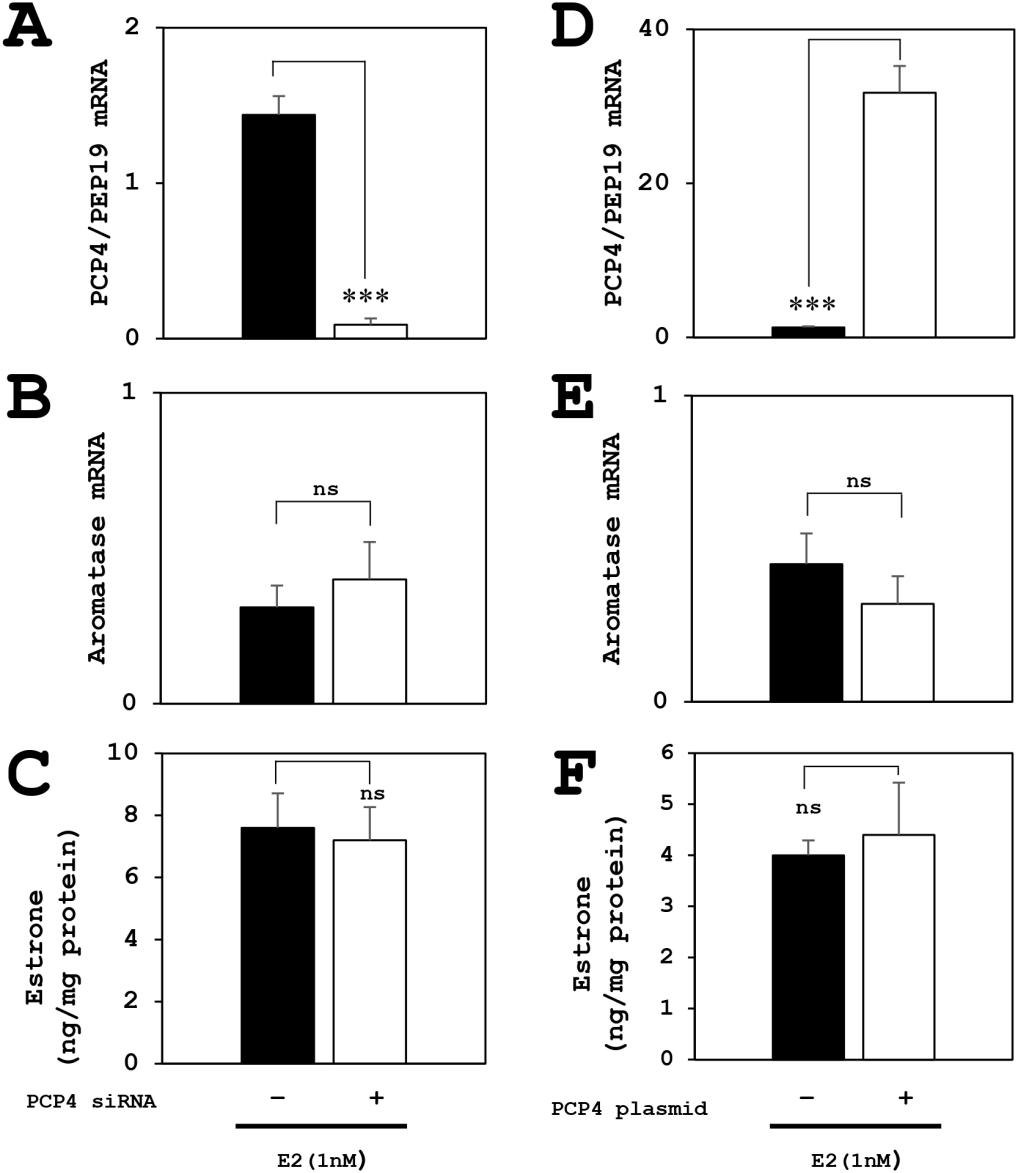
Effects of PCP4/PEP19 on aromatase expression for E1 production in MCF-7 cells under E2 stimulated condition **(A, B, C)** The PCP4/PEP19 knockdown (A) did not decrease the aromatase mRNA expression (B) and E1 production (C) in E2 stimulated conditions. **(D, E, F)** Similarly, The PCP4/PEP19 overexpression (D) did not enhance the aromatase mRNA expression (E) and E1 production (F) in E2 stimulated conditions, either. ^***^ p<0.001 vs without knockdown or overexpression; ns, not significant.

The aromatase mRNA expression was reduced only in SK-BR-3 cells but not in MCF-7 cells after PCP4/PEP19 knockdown (Figure 3A). Conversely, PCP4/PEP19 overexpression by plasmid transfection enhanced the aromatase mRNA expression in SK-BR-3, but not in MCF-7 cells (Figure 3B). MCF-7 cells is known to have a relatively low aromatase activity [18]. Furthermore in the present study, both PCP4/PEP19 knockdown and overexpression did not change the aromatase gene expression and E1 production activity in MCF-7 cells, even cultured in 1 nM E2 supplemented medium (Figure 4).

### Effects of ER knockdown on aromatase expression in MCF-7 cells after PCP4/PEP19 knockdown and overexpression

**Figure 5:**
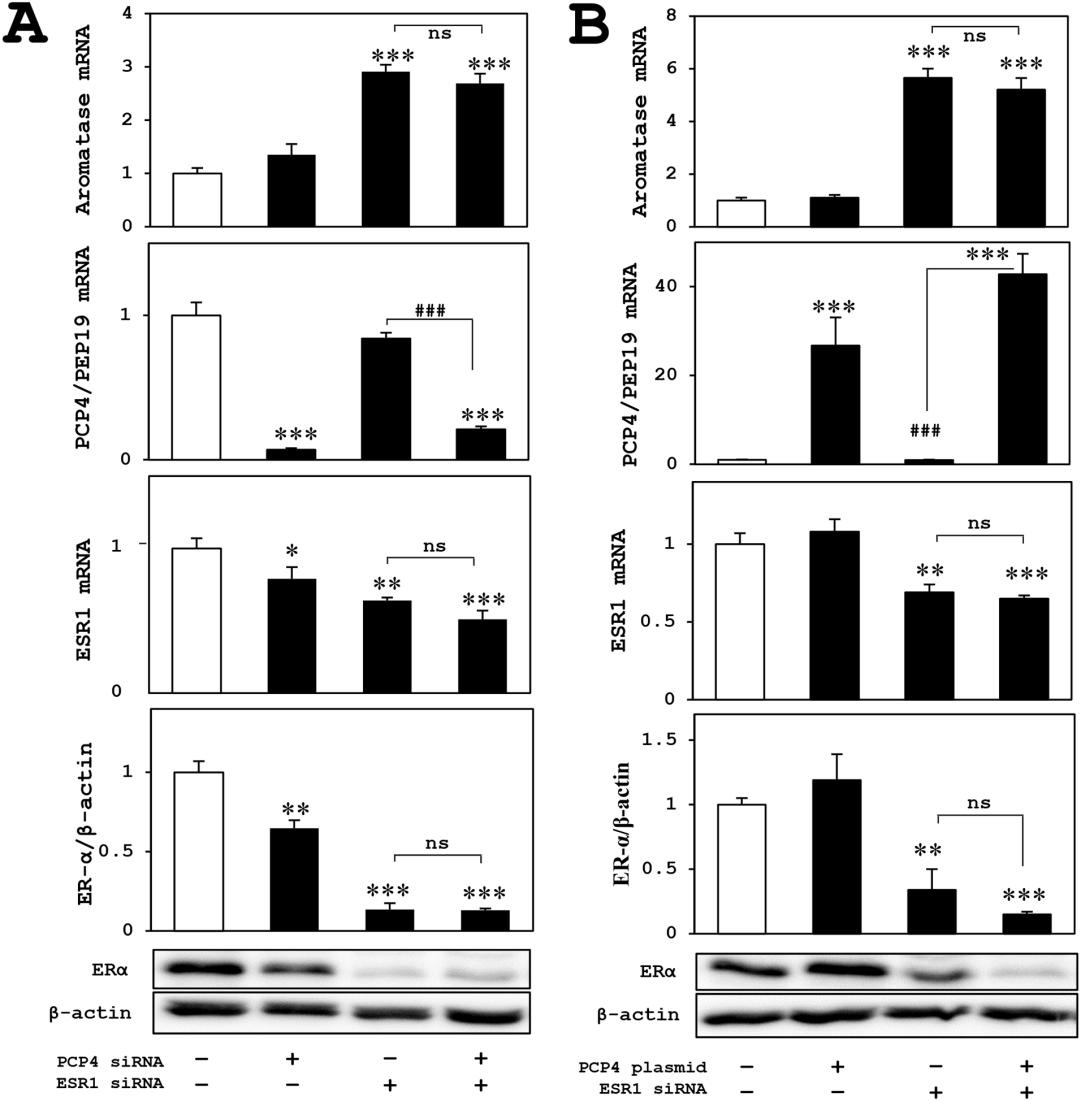
Effects of ER knockdown on aromatase expression in MCF-7 cells after PCP4/PEP19 knockdown and overexpression **(A** and **B)** The aromatase expression of ER knockdowned-MCF-7 cells (upper panels) was not influenced in PCP4/PEP19 knockdown (A) and overexpression (B) with E2 stimulated conditions. The lower 3 panels in (A) and (B) showed mRNA and protein expression of ER and PCP4/PEP19 in each experimental condition. ^*^p<0.05, ^**^p<0.01 and ^***^p<0.001 vs without any genes knockdown or overexpression; ^###^ p<0.001 between indicated conditions; ns, not significant.

In MCF-7 cells, both mRNA (ESR1) and protein (ER-α) expression effectively downregulated by siRNA and the aromatase mRNA expression increased after ESR1 knockdown, which might be a positive feedback mechanism. The enhanced aromatase mRNA expression was not suppressed by PCP4/PEP19 knockdown (Figure 5A) or overexpression (Figure 5B). This indicated that the effects of PCP4/PEP19 on aromatase expression would be very unique in SK-BR-3 cells and independent on ER status. Therefore, measurement of aromatase enzyme activities, promoter specific RT-qPCR and luciferase promoter assay were performed in SK-BR-3 cells for the next experiments.

### Effects of PCP4/PEP19 knockdown and overexpression on aromatase enzyme activity for E1 and E2 production in SK-BR-3 cells

**Figure 6:**
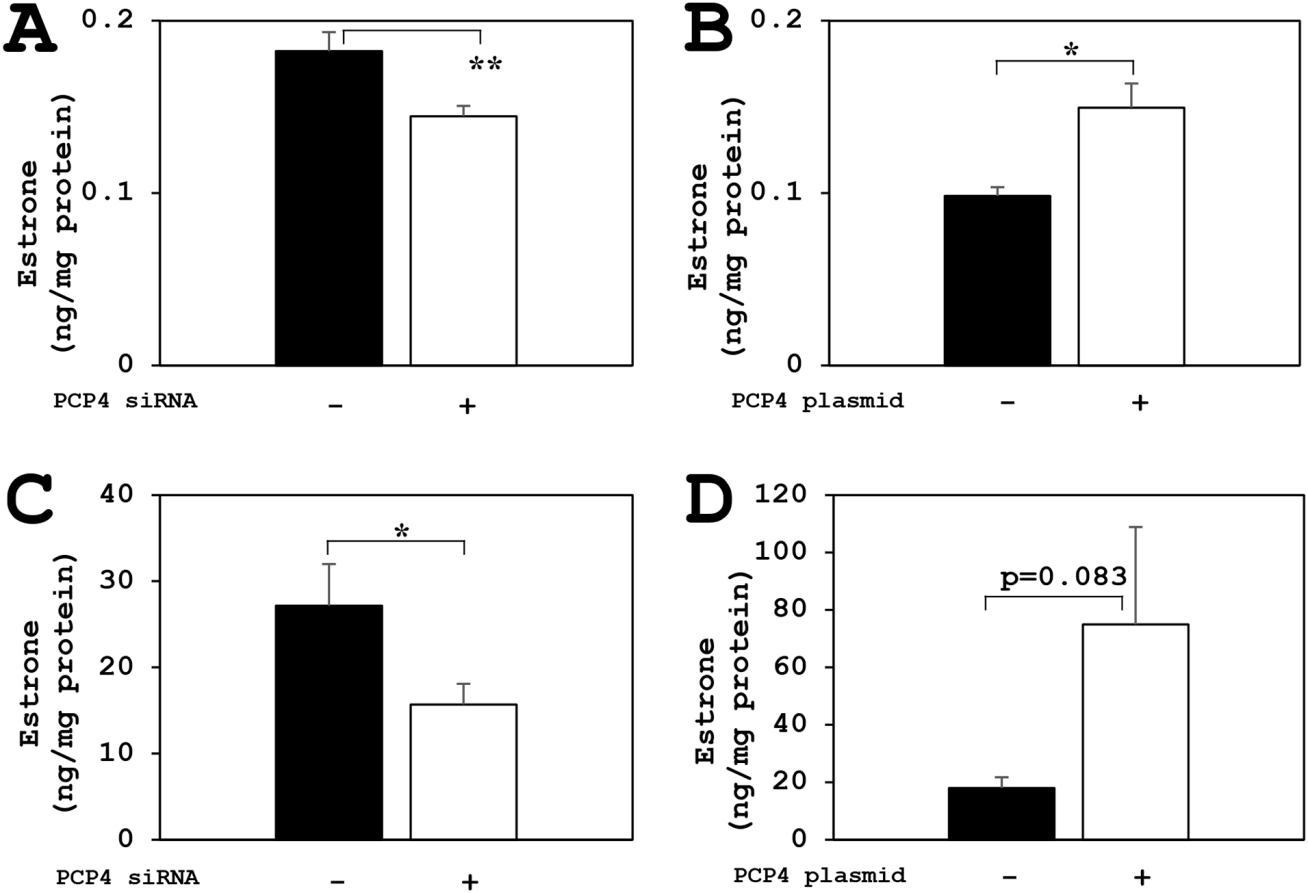
PCP4/PEP19 overexpression and knockdown effects on E1 production in SK-BR-3 cells The aromatase enzyme activities for E1 biosynthesis were measured in both secreted and non-secreted fractions. **(A** and **B)** Secreted fractions of E1 biosynthesis from aromatase activity in SK-BR-3 cells were significantly decreased after PCP4/PEP19 knockdown (A: PCP4 siRNA + as open column) and increased after PCP4/PEP19 overexpression (B: PCP4 plasmid + as open column). **(C** and **D)** Non-secreted fractions of E1 biosynthesis from aromatase activity were also significantly decreased after PCP4/PEP19 knockdown (C: PCP4 siRNA + as open column). E1 production in the cell lysate fractions increased after PCP4/PEP19 overexpression (D: PCP4 plasmid + as open column) with a certain trend toward significance (p=0.083). ^*^p<0.05 and ^**^ p<0.01 vs without knockdown or overexpression.

**Figure 7:**
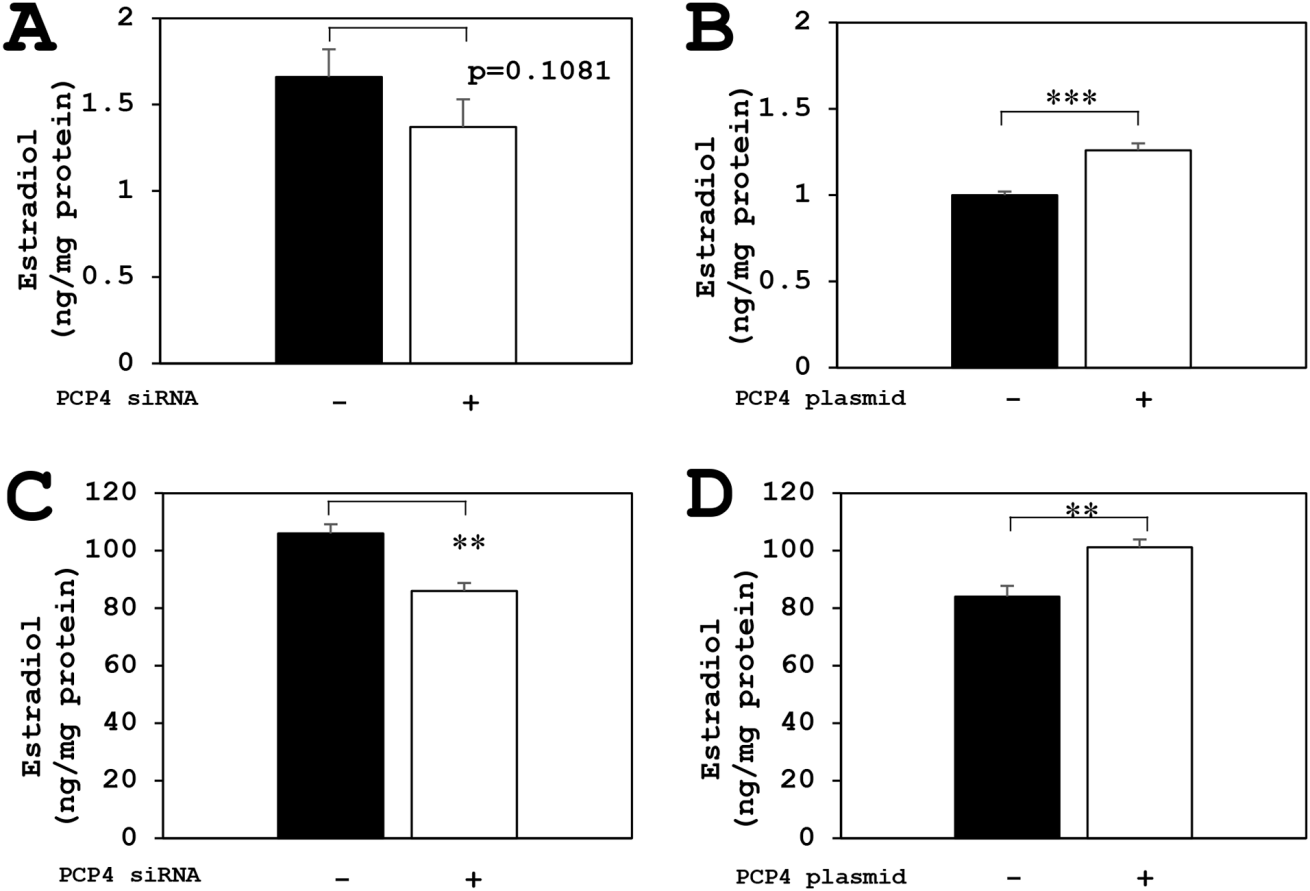
PCP4/PEP19 overexpression and knockdown effects on E2 production in SK-BR-3 cells The aromatase enzyme activities for E2 production were measured in both secreted and non-secreted fractions. **(A** and **B)** Secreted fractions of E2 biosynthesis from aromatase activity in SK-BR-3 cells were significantly increased after PCP4/PEP19 overexpression (B: PCP4 plasmid + as open column), but it showed no significance (p=0.1081) in PCP4/PEP19 knockdown (A: PCP4 siRNA + as open column). **(C** and **D)** Cytoplasmic fractions of E2 biosynthesis from aromatase activity were also significantly decreased after PCP4/PEP19 knockdown (C: PCP4 siRNA + as open column) and increased after PCP4/PEP19 overexpression (D: PCP4 plasmid + as open column). ^**^p<0.01 and ^***^ p<0.001 vs without knockdown or overexpression.

The aromatase enzyme activities for E1 biosynthesis from androstenedione as a substrate were measured by ELISA method. The E1 biosynthetic activities of aromatase in SK-BR-3 cells were decreased after PCP4/PEP19 knockdown in both secreted and intracellular fractions (Figure 6A and 6C). In contrast, after PCP4/PEP19 overexpression, E1 secretion was significantly increased in the medium (Figure 6B). The cytoplasmic E1 fractions showed a borderline significance (p=0.083) after PCP4/PEP19 overexpression (Figure 6D). Similarly, the aromatase activities of E2 production from testosterone were also measured and the E2 biosynthetic activities in SK-BR-3 cells were significantly increased after PCP4/PEP19 overexpression in both secreted and cellular lysate fractions (Figure 7B and 7D). After PCP4/PEP19 knockdown, E2 secretion in the medium was not significantly decreased (p=0.1081) (Figure 7A). In contrast, the E2 in the intracellular fractions showed a significant reduction after PCP4/PEP19 knockdown (Figure 7C). Remarkably, the most amounts of estrogens were stored in the intracellular fractions nearly 100 times more than in the secreted fractions. SK-BR-3 cells express aromatase and produce E1 and E2 which would be possibly upregulated by PCP4/PEP19, especially in intracellular fractions, even though E2 secretion was not significantly reduced by PCP4/PEP19 knockdown.

Since expression of 17β-hydroxysteroid dehydrogenase (interconversion enzyme of E1 and E2), sulfotransferase (catalyzes sulfate conjugation of E1 and E2), and sulfatase (conversion enzyme for sulfated E1/E2 to free E1/E2) was not changed after PCP4/PEP19 knockdown and overexpression (data not shown), the increased E1 and E2 production would be, at least in part, derived from the increased expression of aromatase in SK-BR-3 cells.

### Promoter specific RT-qPCR of aromatase mRNA expression in SK-BR-3 cells

**Figure 8:**
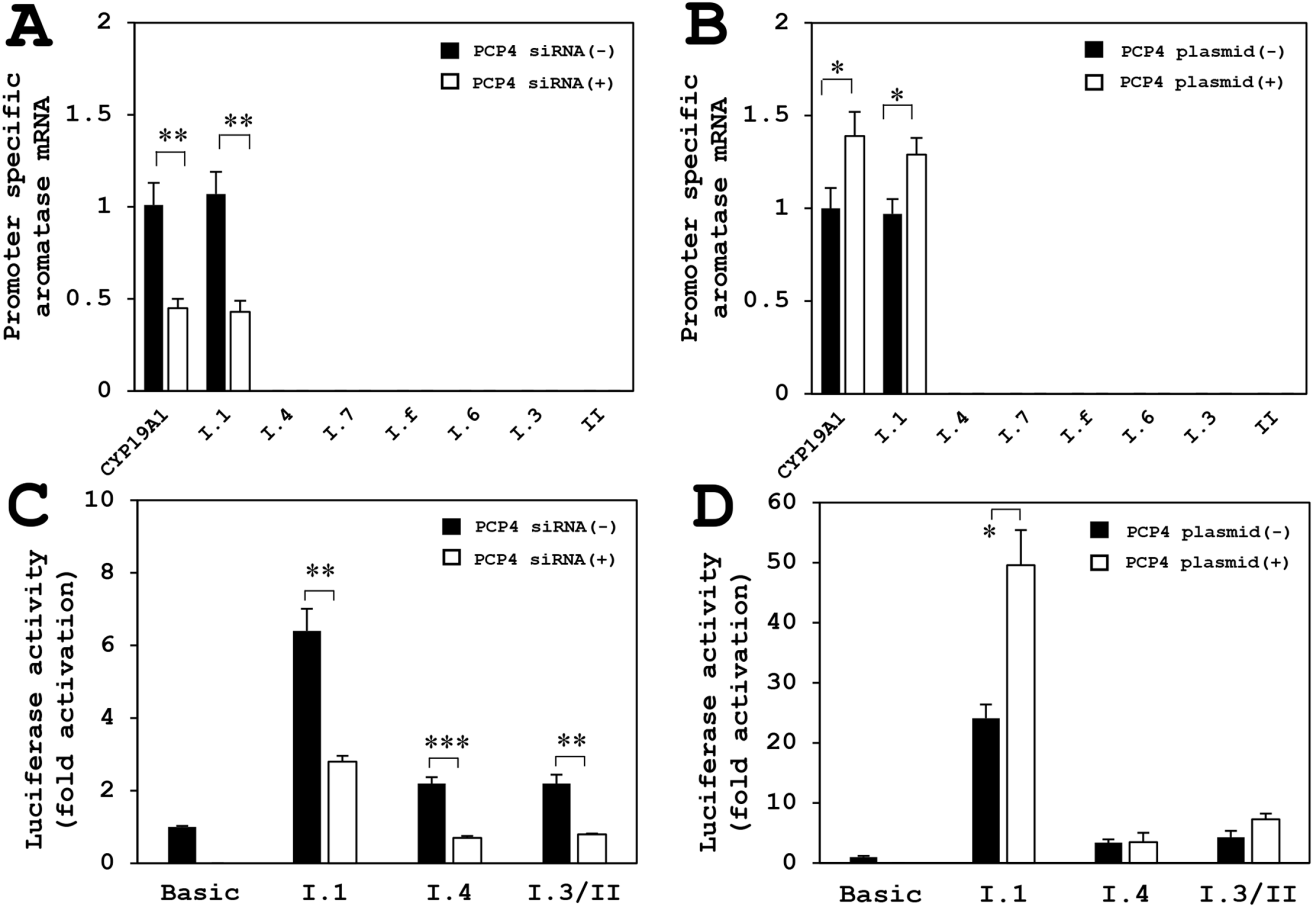
Promoter specific RT-qPCR for aromatase mRNA expression after PCP4/PEP19 knockdown and overexpression (A, B), and luciferase reporter assay using promoter regions PI.1, PI.3/II and PI.4 in SK-BR-3 cells (C, D) **(A)** The aromatase mRNA expression by promoter non-specific RT-qPCR amplifying coding region (CYP19A1) was decreased after PCP4/PEP19 knockdown (PCP4 siRNA + as open column) as shown in Figure 3A. Promoter specific RT-qPCR showed decreased aromatase mRNA expression from the PI.1 region after PCP4/PEP19 knockdown (I.1). The mRNA transcription from PI.3, PI.4, PI.6, PI.7 and PII regions was very low (I.3, I.4, I.6, I.7 and II). **(B)** The mRNA expression of coding region of aromatase (CYP19A1 as shown in Figure 3B) and transcript from PI.1 were increased after PCP4/PEP19 overexpression (I.1) (PCP4 plasmid + as open column). The mRNA expression from PI.3, PI.4, PI.6, PI.7 and PII regions were not detected in our experimental conditions (I.3, I.4, I.6, I.7 and II). **(C)** The transcriptional activities from the promoter PI.1 region was significantly higher than those from PI.3/II and PI.4 promoter regions. These activities were downregulated by PCP4/PEP19 mRNA silencing (PCP4 siRNA + as open column). **(D)** After PCP4/PEP19 was overexpressed by plasmid transfection (PCP4 plasmid + as open column) in SK-RB-3 cells, the transcriptional activities from promoter PI.1 region were significantly enhanced but not those from the PI.3/II and PI.4. The luciferase activities from each construct were normalized by protein concentration and presented as fold activation relative to those from pGL_3_ basic vector (Basic). ^*^p<0.05, ^**^p<0.01 and ^***^p<0.001 vs without knockdown or overexpression.

The aromatase mRNA expression, analyzed by promoter non-specific RT-qPCR using paired primers annealing to coding region of the aromatase gene, was decreased by PCP4/PEP19 knockdown (Figure 8A, CYP19A1). Promoter specific RT-qPCR showed that aromatase mRNA expression from the promoter region PI.1 was significantly reduced after PCP4/PEP19 knockdown (Figure 8A, I.1), while the mRNA transcription from PI.3, PI.4, PI.6, PI.7, PI.f and PII promoter regions was very low in SK-BR-3 cells (Figure 8A). The mRNA expression from the CYP19A1 and PI.1 were increased after PCP4/PEP19 overexpression (Figure 8B, CYP19A1 and PI.1). Even in the overexpression of PCP4/PEP19, the aromatase expression from PI.3, PI.4, PI.6, PI.7, PI.f and PII regions were not detected in our experimental conditions (Figure 8B).

### Luciferase reporter assay using promoter regions PI.1, PI.3/II and PI.4

The transcriptional activities from the promoter PI.1 region was markedly higher than those from the promoter PI.3/II and PI.4 regions, and these activities were downregulated by PCP4/PEP19 mRNA silencing (Figure 8C). When PCP4/PEP19 was overexpressed in SK-RB-3 cells, transcriptional activities from the promoter PI.1 region were significantly enhanced but not those from the promoter PI.3/II and PI.4 regions (Figure 8D). Together with the results obtained from the promoter specific qPCR analysis, possible dominant motif(s) of aromatase gene transcription by PCP4/PEP19 would be located in the PI.1, although it is not explainable why PCP4/PEP19 knockdown reduced the promoter activities but PCP4/PEP19 overexpression did not enhance those from the PI.4 and I.3/II.

### Immunolocalization and semi-quantitative analysis of PCP4/PEP19 and aromatase in human breast cancer tissues in terms of ER status

**Figure 9:**
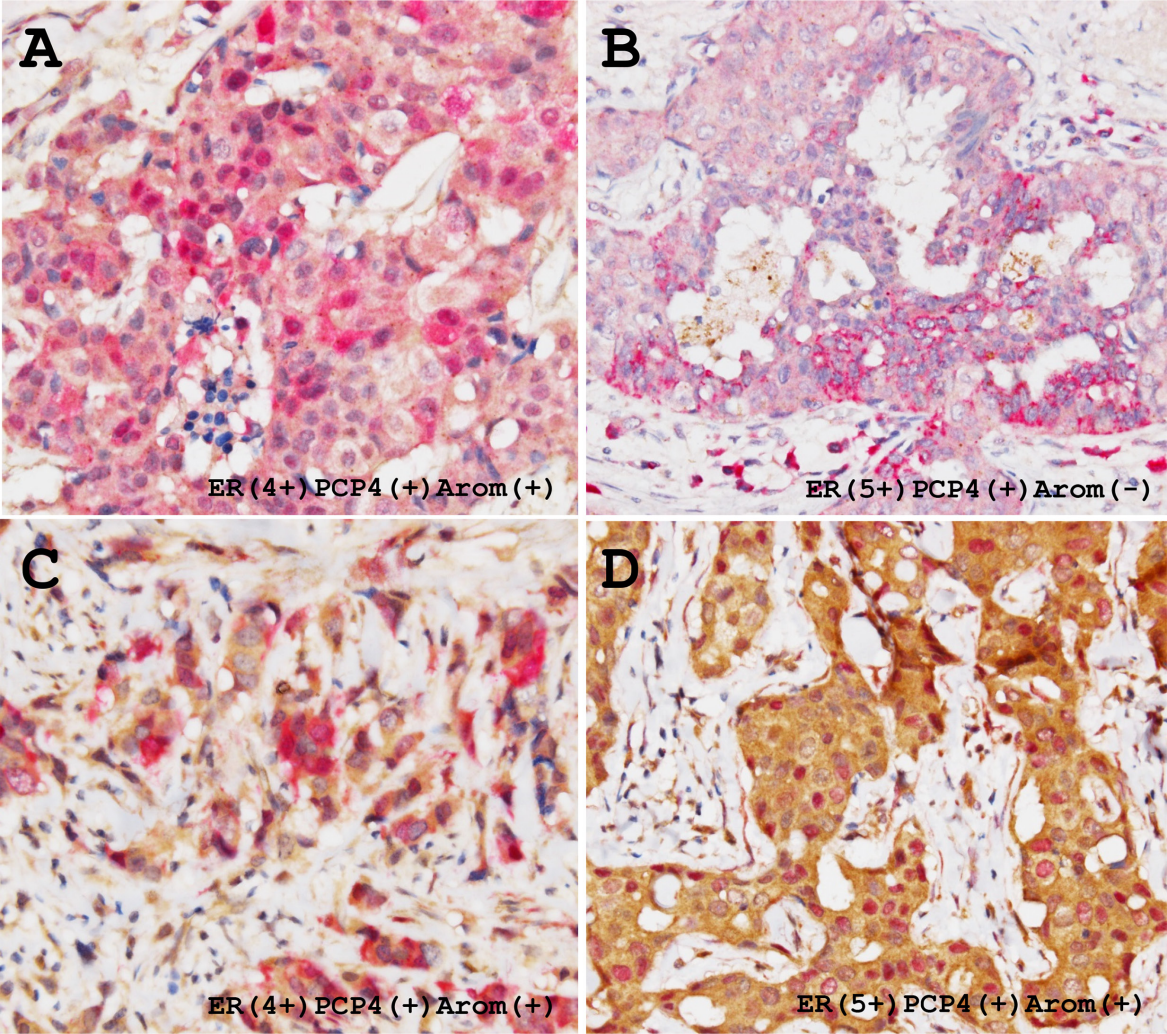
Representative immunohistochemical demonstration of PCP4/PEP19 and aromatase expression in ER-positive human breast cancer tissues Thirty four % cases of the breast cancers were positive for both PCP4/PEP19 and aromatase. PCP4/PEP19 positive cells (red) and aromatase positive cells (brown) are mixed in the breast cancer tissues, which results in a mosaic pattern **(A** and **C)**. Some carcinoma cells express both PCP4/PEP19 and aromatase, having red-brown colored cytoplasm (A, C), while some were only positive for PCP4/PEP19 **(B)**. In some cases, nuclear localization of PCP4/PEP19 was observed in the aromatase-positive breast cancer cells **(D)**.

Among 44 ductal carcinomas, 75% and 52% of cases were positive for aromatase and PCP4/PEP19, respectively. The ductal carcinoma cells expressed both PCP4/PEP19 and aromatase in 34 % cases (15/44 cases). Expression status of ER was not correlated to that of PCP4/PEP19 and aromatase (Table 1). The expression pattern was highly heterogeneous; some tumor cells were positive for either PCP4/PEP19 or aromatase and some were positive for both. The PCP4/PEP19 was detected in both the cytoplasm and nuclei and aromatase was observed only in the cytoplasm (red, PCP4/PEP19; brown, aromatase, Figure 9). Among these PCP4/PEP19 and aromatase double positive cancer tissues (15 cases), aromatase expression was diffusely detected and some portions were positive for PCP4/PEP19. Positive percentage fraction of PCP4/PEP19 relative to that of aromatase was 69.2% in ER^+^ breast cancers, but only 22.5% in ER^-^ cancers (p=0.011) (Table 2). PCP4/PEP19 positive cells were widely detected in cells ranging ER 5+ to ER 0 cases (Table 2). In some cases, stromal cells were also positive for PCP4/PEP19 irrespective of ER status as previous indicated [19] (Figure 9B).

**Table 1:** Expression of PCP4/PEP19 and aromatase in relation to ER status

	PCP4(-)	PCP4(+)	total		Arom(-)	Arom(+)	total
ER^-^	2	7	9	ER(-)	3	6	9
ER^+^	19	16	35	ER(+)	8	27	35
total	21	23	44	total	11	33	44

	PCP4(+)Arom(-)	PCP4(+)Arom(+)	PCP4(-)Arom(-)	PCP4(-)Arom(+)	total
ER^-^	3	4	0	2	9
ER^+^	5	11	3	16	35
total	8	15	3	18	44

ER, estrogen receptor; Arom, aromatase; PCP4, PCP4/PEP19

No significant correlation among these expression.

**Table 2:** PCP4/PEP19 and ER categories in double positive cases

ER^+^ cases		ER^-^ cases	
ER	%PCP	ER	%PCP
5+	100	1+	46
5+	100	0	30
5+	100	0	9
5+	100	0	5
5+	98		
5+	82		
5+	56		
5+	36		
4+	41		
4+	32		
4+	16		
Mean ± SE	69.2 ± 10.0	p = 0.011	22.5 ± 9.6

ER, estrogen receptor; PCP4, PCP4/PEP19

## DISCUSSION

In this study, we demonstrated that the PCP4/PEP19 could induce aromatase expression in ER^-^ SK-BR-3 human breast cancer cells. The results showed that the PCP4/PEP19 upregulates transcriptional activity from promoter PI.1 of the aromatase gene. In general, the promoter PI.1 has been reported to be placental specific and its higher activity results in elevated blood levels of estrogens in pregnant women [9]. The utilization of PI.1 promoter would not be common in breast cancer cells, but SK-BR-3 cells are known to utilize the PI.1 promoter for aromatase expression [20]. Moreover, after ER-α is transiently overexpressed in ER^-^ SK-BR-3 cells, E2 stimulates the cells to enhance the transcriptional activity from the promoter PI.1 [18]. We showed that ER knockdown did not change the expression levels of aromatase in MCF-7 cells, irrespective of PCP4/PEP19 expression levels. Taken together, these indicate that the aromatase expression in SK-BR-3 cells would be, at least in part, dependent on the PI.1 promoter activity irrespective of ER-α expression status. The aromatase PI.1 promoter utilization is very unique features in SK-BR-3 cells and therefore, SK-BR-3 cells would be valuable to study a paracrine mechanism of estrogens biosynthesis and effects of bioactive factors on cell-specific promoter activity, although main promoter utilization of aromatase gene expression is PI.3, PI.4 and PII in the human breast cancer tissues and cell lines including MCF-7, T-47D and MDA-MB-231 cells [21].

Our previous study demonstrated that the PCP4/PEP19 expression is induced by E2 in ER^+^ MCF-7 human breast cancer cells and is constitutively higher in ER^-^ SK-BR-3 cells [13]. However, aromatase expression in wild type MCF-7 cells was not influenced by decreased or increased expression levels of PCP4/PEP19, which were introduced by knockdown or overexpression, respectively. Thus, the PCP4/PEP19 is acting to induce aromatase expression only in SK-BR-3 cells but not in MCF-7 cells. Whereas MCF-7 cells, which have relatively lower aromatase activity, are induced to express aromatase expression by ER-α overexpression and E2 treatment [18, 20]. All these suggest that the expression of PCP4/PEP19 is not connected to the induction of aromatase expression in MCF-7 cells, although the PCP4/PEP19 promotes cancer cell proliferation and survival mediated through its anti-apoptotic effects and also increases cancer cell migration and invasion in both MCF-7 and SK-BR-3 cells [13, 16]. The functions of PCP4/PEP19 would be divergent among the human breast cancer cell lines.

The mode of estrogens action is classified into two mechanisms; one is mediated through a nuclear translocation of E2-ER complex, which binds to the enhancer elements and transactivates estrogens target genes, representing genomic action. Another mechanism is referred to as a non-genomic action, by which E2 binds to membrane or cytosolic ER to crosstalk with other signaling pathways such as epidermal growth factor and mitogen-activated protein/extracellular signal-regulated kinase pathway [18, 22, 23]. Actually, E2-induced aromatase expression in ER-α overexpressed SK-BR-3 cells is explained by this non-genomic action [18]. In the present study, however, the PCP4/PEP19 could activate the aromatase PI.1 promoter activity even in wild type ER^-^ SK-BR-3 cells. Thus, the PCP4/PEP19 signaling pathway for aromatase gene regulation would be independent on both genomic and non-genomic action of E2. The promoter PI.1 of human aromatase gene includes several known distal motifs binding for CCAAT/enhancer-binding protein family, retinoid X receptor, trophoblast-specific element binding factor, AP-1 and potential binding site for Sp1 but not classic estrogen-responsive element [24–27]. The promoter PI.1 region might include an element(s) linking to still unknown PCP4/PEP19 signaling pathway, which would be working even in ER^-^ SK-BR-3 cells. The transcriptional regulation of aromatase gene mediated via PI.1 by PCP4/PEP19 might be caused by genomic or epigenomic changes occurred in SK-BR-3 cells during the process of cell line establishment. Exact process for the aromatase gene expression by PCP4/PEP19 still remains to be clarified in the next study.

Human breast cancer tissues consist of heterogeneous cancer cell population. This is known as intratumoral heterogeneity, in which paracrine interactions between ER^+^ and ER^-^ cancer cells promote tumor progression via signaling crosstalk [28, 29]. For example, intratumoral production of estrogens by aromatase gene expression in some cancer cell population would be an autocrine or paracrine source of estrogens for ER^+^ cancer cell survival and proliferation [30, 31]. Hence, locally supplied estrogens by aromatase gene upregulation in PCP4/PEP19 expressed ER^-^ cancer cell population, like SK-BR-3 cell phenotype, would stimulate ER^+^ cancer cell population to enhance proliferation, migration and invasion, like MCF-7 cell phenotype. Actually, both PCP4/PEP19 and aromatase were detected in both ER^+^ and ER^-^ human ductal carcinomas, in which ER expression categories were ranging ER 5+ to ER 0. It is also worth noting that the nuclear localization of PCP4/PEP19 has been demonstrated in some cells, indicating a possibility that PCP4/PEP19 enhances aromatase expression as a nuclear transcriptional factor.

In summary, PCP4/PEP19 upregulates aromatase gene expression mediated through PI.1 promoter transcriptional activity in ER^-^ SK-BR-3 but not in ER^+^ MCF-7 human breast cancer cells. In addition to the PCP4/PEP19 functions inhibiting apoptosis and increasing migratory and invasive activities [13, 16], the present study indicates a new function, by which PCP4/PEP19 would enhance breast cancer cell proliferation by promoting paracrine estrogen-signaling via aromatase expression. Since the intratumoral estrogen-production correlates with prognosis of the breast cancer patients, additional study to clarify the relation between PCP4/PEP19 and aromatase expression would be very important to further understand the breast cancer proliferation and progression.

## MATERIALS AND METHODS

### Cells and cell culture

Human breast cancer MCF-7 cells were obtained from RIKEN BioResource Center (Tsukuba, Japan). Human breast cancer SK-BR-3 cells were purchased from American Type Culture Collection (Rockville, MD). The MCF-7 and SK-BR-3 cells were maintained in minimal essential medium (MEM) (Sigma, St. Louis, MO) and McCoy’s 5A (Sigma) supplemented with 2 mM glutamine, 100 U/mL penicillin, 100 μg/mL streptomycin, and 10% fetal bovine serum (FBS). These cells were maintained at 37°C with 95% air and 5% CO_2_. For removal of steroid hormones from FBS, a 5% charcoal and 0.5% dextran (Sigma) suspension in FBS was incubated at 37°C for 1 hr with shaking. The suspension mixture was then centrifuged at 2,500 rpm for 20 min, and the supernatant was filtered through a 0.2 μm filter. For experiments, unless otherwise noted, the SK-BR-3 cells were cultured in 10% FBS supplemented medium and MCF-7 cells were cultured in 5% charcoal-stripped FBS. In some experiments, MCF-7 cells were cultured in the medium containing 1 nM E2.

### Plasmid construction

For PCP4/PEP19 overexpression experiments, PCP4 expression vector driven by CMV promoter was purchased from OriGene Technologies (Rockville, MD). Luciferase reporter construct containing human aromatase promoters PI.4 was kindly gifted from Dr. Colin Clyne in Prince Henry’s Institute of Medical Research (Victoria, Australia) [32]. The PI.1, PI.6, PI.7, PI.f and PI.3/II has been generated as previously [33–37]. The pGL_3_ basic vector (Promega, Maddison, WI) was used as a control for luciferase assay.

### Transfection experiments

For knockdown experiments, pre-designed siRNAs were used for knockdown of PCP4/PEP19 (ID: HSS181928), ESR1 (ID: VHS40913) and Stealth RNAi siRNA Negative Control was used as negative control (Thermo Fisher Scientific, Waltham, MA). The cells were transfected with siRNAs using Lipofectamine RNAiMAX in Opti-MEM I (Thermo Fisher Scientific) according to the manufacturer’s instructions. For the plasmid transfection, ScreenFect A plus (Wako Pure Chemical, Osaka, Japan) was used according to the manufacturer’s instructions.

### Quantitative RT-PCR analysis of gene expression

Total RNA was extracted using ReliaPrep RNA Cell Miniprep System (Promega) and was converted into cDNA using a High Capacity RNA-to-cDNA kit (Thermo Fisher Scientific). The cDNA was analyzed by LightCycler 480 (Roche Diagnostics, Basel, Switzerland). Each sample was analyzed in triplicate in separate wells for the target and reference genes using the following primers listed in Table 3. The average of three threshold cycle values for the target and reference genes was calculated and analyzed with the comparative Ct method.

**Table 3:** Primers and probes for PCR of CYP19A1, PCP4/PEP19 and ESR1 mRNA analysis

	Target genes	Primer sequence (5’ – 3’)	References
Taqman assay	CYP19A1	Assay ID: Hs00903411_m1	Thermo Fisher Scientific
	PCP4/PEP19	Assay ID: Hs01113638_m1	Thermo Fisher Scientific
	ESR1	Assay ID: Hs01046816_m1	Thermo Fisher Scientific
	18S rRNA	Assay ID: Hs99999901_s1	Thermo Fisher Scientific
Promoter specific RT-qPCR	CYP19A1 (F)	TGG AAA TGC TGA ACC CGA TAC	-
	I. 1 (F)	TGT GCT CGG GAT CTT CCA GAC	20
	I. 3 (F)	GGG CTT CCT TGT TTT GAC TTG TAA	20
	I. 4 (F)	GTG ACC AAC TGG AGC CTG	20
	I. 6 (F)	CAC AGC AGA ACC AGC ACA TCA	21
	I. 7 (F)	GGC TCC ATC TAC AAG GAT GA	18
	I. f (F)	GAA AAG CCA CCT GGT TCT TA	22
	II (F)	CTC TGA AGC AAC AGG AGC TAT AGA T	20
	Reverse	CCA ATT CCC ATG CAG TAG CCA	-
	Probe	[FAM] CTGCTGCCACCATGCCAGTCCTG [TAMRA]	-

### Quantification of estrone and estradiol levels

Aromatase catalyzed conversions of androstenedione to estrone (E1) and that of testosterone to estradiol (E2) were measured for aromatase activities. Cells were seeded at 1.5 x 10^5^ cells/well in 24-well plates. After transfection of PCP4/PEP19 expressing plasmid vectors for overexpression or siRNA for PCP4/PEP19 mRNA silencing, the cells were incubated for 48 hr with 100 nM 4-androstene-3, 17-dione (Tokyo Chemical Industry Co., Ltd., Tokyo, Japan), or 100 nM testosterone (Nacalai tesque, Inc., Kyoto, Japan), respectively as substrates. Since testosterone is converted to dihydrotestosterone by 5 α-reductase, 5 α-reductase inhibitor finasteride (10 μM) was added to the medium when measuring E2 concentration. The cells were harvested by trypsinization, and lysed with 1 mM EDTA with sonication. E1 and E2 concentrations of conditioned media and cell lysate were measured by ELISA (YK180 Estrone EIA kit and YK170 17 β-Estradiol EIA Kit, Yanaihara Institute, Shizuoka, Japan) according to manufacturer’s instructions. The values were normalized by protein concentration of the cell lysates.

### Luciferase reporter assays

The cells were seeded at the density of 1.0 x 10^5^ cells/well in 24-well plates, and reporter plasmids were transfected on the next day. At 48 hours after transfection, 100 nM 4-androstene-3, 17-dione was added to medium, and the cells were incubated for another 48 hours. The cells were lysed, and the luciferase activities were measured using PicaGene reagent (TOYO B-Net, Tokyo, Japan). The luciferase bioluminescence was quantified by a PHELIOS luminometer (ATTO, Tokyo, Japan) and the values were normalized by protein concentration of the cells. The protein concentration was determined using coomassie protein assay reagent (Thermo Fisher Scientific).

### Western blot analysis

Cells were washed with PBS and were precipitated with 10% trichloroacetic acid on ice for 30 min. The precipitates were washed with cold PBS and dissolved in cold lysis buffer (50 mM Tris-HCl [pH 6.8], 2% SDS, 10% glycerol, 6% 2-mercaptoethanol and 0.01% bromophenol blue). The lysates were then analyzed using tris/tricine or tris/glycine gels, and transferred to PVDF membranes. The membranes were blocked with 5% non-fat dried milk in TBS (pH 7.6) with 0.1% Tween 20 for 1 hr and then incubated overnight at 4°C with primary antibody diluted in Can Get Signal solution 1 (Toyobo, Osaka, Japan) and with a horseradish peroxidase conjugated goat anti-rabbit antibody (Cell Signaling Technology) for 1 hr at room temperature. Anti-PCP4/PEP19 polyclonal rabbit antibody was purchased from Sigma. Antibodies against estrogen receptor-α (ER-α) and beta-actin (β-actin) were obtained from Cell Signaling Technology (Danvers, MA). Anti-phospho-ER-α^Ser167^ was from Biorbyt Ltd. (Cambridge, UK). Protein expression was detected with SuperSignal West Pico chemiluminescent substrate or SuperSignal West Femto Maximum Sensitivity Substrate (Thermo Fisher Scientific). Densitometry analysis was performed using CS Analyzer 3.0 (ATTO).

### Immunohistochemistry

Formalin fixed paraffin embedded tissue sections were used for immunohistochemical detection of PCP4/PEP19 and aromatase expression in human invasive ductal carcinoma cases resected at Hakuaikai Sagara Hospital. Anti-PCP4/PEP19 polyclonal rabbit antibody was purchased from Sigma and anti-aromatase monoclonal mice antibody was from Bio-Rad Laboratories (Hercules, CA) [38, 39]. For double immunostains, Double Stain Polymer Detectin Kit was used (Biocare Medical, CA). The breast cancer cases, in which more than 25% of tumor areas were positive for either PCP4/PEP19 or aromatase, were considered as positive expression. Since aromatase diffusely expressed in almost all tumor cells in PCP4/PEP19 and aromatase double positive cases, percentage fraction of PCP4/PEP19 positive cells relative to aromatase positive cells were calculated. ER-α status was subclassified into 6 categories; 5+ for more than equal to 90% of tumor nuclei were positive, 4+ for 50 to 90% positive, 3+ for 10 to 50% positive, 2+ for 1 to 10%, 1+ for less than 1% positive nuclei, 0 for no positive cells. ER expression status of 1+ and 0 were classified into ER^-^ cancers, and other categories were classified into ER^+^ cancers.

### Statistics

All experiments were performed at least 3 to 6 times independently and all data are presented as mean ± SE. Statistical significance was determined by unpaired one-tailed Student's *t* or chi-square test and p < 0.05 was considered statistically significant.
